# Epithelial-mesenchymal crosstalk: the scriptwriter of craniofacial morphogenesis

**DOI:** 10.3389/fcell.2024.1497002

**Published:** 2024-11-08

**Authors:** Junjie Lu, Bo Peng, Wenyi Wang, Yi Zou

**Affiliations:** ^1^ School of Life Science and Technology, Jinan University, Guangzhou, China; ^2^ Institute for Environmental and Climate Research, Jinan University, Guangzhou, China

**Keywords:** epithelial-mesenchymal interaction, craniofacial development, cleft lip/palate, neural crest cells, FEZ

## Abstract

Epithelial-mesenchymal interactions (EMI) are fundamental mechanisms in regulating development and organogenesis. Here we summarized the signaling mechanisms involved in EMI in the major developmental events during craniofacial morphogenesis, including neural crest cell induction, facial primordial growth as well as fusion processes. Regional specificity/polarity are demonstrated in the expression of most signaling molecules that usually act in a mutually synergistic/antagonistic manner. The underlying mechanisms of pathogenesis due to disrupted EMI was also discussed in this review.

## 1 Introduction

Orofacial clefts (OFC), including cleft lip and cleft palate, are one of the most common birth defects with an approximate incidence of 1.7 per 1,000 live births, vary with ethics, territories, prenatal exposure as well as socioeconomic status (SES) (Clark et al., 2003; Mossey et al., 2009; Mossey and Modell, 2012). Clefts can be presented as isolated malformations or as part of the overlapping manifestations of syndromes with distinct entities. Understanding of lip and palate development is essential for the mechanogenesis, prevention, treatment, and prognosis of OFC. Despite the discrepancies in facial anatomies and tissue origins, vertebrates are highly conserved in the early craniofacial development, which relies on the precisely regulated outgrowth of five facial prominences–the frontonasal prominence (FnP) that develop into the medial nasal (MnP) and lateral nasal prominences (LnP), the bilaterally paired maxillary (MxP) and mandibular prominences (MdP), and their subsequent fusion (Jiang et al., 2006). These facial processes, including the palatal shelves, are covered by epithelial cells of ectodermal origin, with the underneath mesenchyme derived primarily from the neural crest (Jiang et al., 2006; Li et al., 2017). The cranial neural crest cells, a stem cell population that emigrated from the borders between non-neural ectoderm and the closing neural tube, migrate ventrally via well-defined routes and populate the mesenchyme of the facial prominences, where they eventually contribute to the formation of craniofacial bone and cartilage, connective tissues and peripheral neurons and glia (Roth et al., 2021). Despite the high versatility in generating a vast range of cell types, the neural crest cells proliferate, migrate and acquest cell fate according to intrinsic programming as well as to constantly changed extrinsic instructions, including reciprocal interactions between NCC and the adjacent cells as well as chemotactic cues in their microenvironment (Erickson et al., 2023). The surface ectoderm outlining embryonic facial prominences acts as the epithelial signaling center of morphogenic instructions, which are bidirectional processes as the expression of agonists/antagonists by neural crest cells are necessary for the maintenance of pattern of expression of morphogenic signaling molecules in the epithelium (Marchini et al., 2021). Proliferation and directed expansion of mesenchymal cells depend on signals from the epithelium, and signals from the mesenchyme influence epithelial cell development (Richman and Tickle, 1989; Francis-West et al., 1998; McGonnell et al., 1998). These interactions involve many intercellular signaling pathways and transcription factors, including sonic hedgehog (Shh), fibroblast growth factor (Fgf), bone morphogenetic protein (Bmp)/transforming growth factor-β (Tgf-β), wingless-type MMTV integration site (Wnt), retinoic acid (RA), and others (Jiang et al., 2006; Li et al., 2017; Atukorala and Ratnayake, 2021; Won et al., 2023).

There are numerous reviews on the craniofacial development and the pathogenesis of OFC from genetic and cellular perspectives (Reynolds et al., 2019; Reynolds et al., 2020; Hammond and Dixon, 2022). However, although EMI are essential processes underlying embryonic morphogenesis such as limb development, odontogenesis and organ formation, an updated comprehensive summary of the epithelial-mesenchymal crosstalk in regulating cranial neural crest cell differentiation and craniofacial development is not available. In this narrative review, after a brief overview of the early craniofacial development, we focused on the mutual communications between the epithelium and mesenchyme in respect to the fundamental cellular events, including NCC proliferation and migration, NCC differentiation, fusion, and epithelial-mesenchymal transition (EMT) during craniofacial formation as well as how disruption in these processes led to malformations.

## 2 Early craniofacial development and morphogenesis

Mammalian heads are distinctive covariation structures that are determined by integrated variations in fundamental developmental processes including neural crest migration/proliferation/patterning, facial process fusion, mesenchyme condensation/differentiation, cartilage/bone formation, brain growth and muscle-bone interaction, as well as the entire somatic development (Carlson and Byron, 2008). These processes arise in a developmental time-order from early after conception to adolescence. Despite the vast interspecies phenotypic differences, the early craniofacial development is highly conserved and for this reason, most of our knowledge about the molecular mechanisms have obtained from using mouse as one of the model organisms (Tamarin and Boyde, 1977; Gritli-Linde, 2008; 2012). Craniofacial morphogenesis starts approximately from the fourth week of human embryonic development (correspondent to E8.5 in murine embryogenesis) with the cranial neural crest cells (CNCC) delaminated from the edge of dorsal neural tube through the process of EMT (O'Rahilly and Müller, 2007; Minoux and Rijli, 2010; Simões-Costa and Bronner, 2015; Soldatov et al., 2019). These CNCC then migrate ventrally to populate the mesenchyme underneath the surface ectoderm of the frontonasal process and the 1^st^ branchial arch, which develop into the maxillary and mandibular processes at fifth week of human and at E9.5 of mouse gestation, respectively (Yoshida et al., 2008; Simões-Costa and Bronner, 2015; Soldatov et al., 2019). By this stage, the facial primordium consisting of five separate epithelium-covered prominences surrounding the invaginated stomodeum can be easily observed, with the FnP at ventromedial, a pair of MxPs and a pair of MdPs at the lateral sides. At stage 14 (33 days after fertilization) of human embryogenesis, equivalent to E10.5 in mouse embryo, the FnP divides into paired MnPs and LnPs by invaginating nasal placodes as well as the proliferating primordial mesenchymal cells (Jiang et al., 2006). The close contact between approaching MnPs, LnPs and MxPs leads to their fusion at the lambdoidal junction at the nasolacrimal groove by stage 16 (E11.0 in mouse embryogenesis) (Jiang et al., 2006).The epithelial seam of opposing prominences eventually breakdown to ensure a confluent mesenchyme covered by a continuous sheath of epithelium by stage 19 (mouse E12.5) (Jiang et al., 2006; Gritli-Linde, 2008; Minoux and Rijli, 2010). The convergence of MnPs in the midline and their inward growth into oral cavity generate the primary palate, which fuses with the secondary palate developed from MxPs starting from approximately E11.5 in mouse (Yu et al., 2017). The palatal shelf grows bilaterally from the inner side of the MxP and extends antero-posteriorly along the lateral walls of the oropharynx. In mice, starting at E11.5, the palatal shelves flanking the tongue grow vertically in the oral-nasal cavity, followed by elevation to a horizontal position above the tongue within a narrow time frame between E14.0-E14.5 (Yu et al., 2017). Further polarized growth results in the approach of the contralateral palatal shelf, which then adheres along its medial edge epithelia (MEE) to form a medial epithelial seam (MES). The gradual disappearance of the MES allows the palatal shelf to fuse along the midline (Gritli-Linde, 2007; 2008). The synchronized growth and fusion of facial primordia are precisely controlled by both genetic and environmental regulations, and perturbations in which may result in abnormalities including orofacial clefts.

## 3 Epithelial-mesenchymal interaction in the specification, delamination and migration of CNCCs

The vertebrate unique neural crest cells (NCCs) arise along the entire length of neural tube at the border between neural and non-neural ectoderm, where they subjected to integrated signals from neural, non-neural ectoderm, and the mesoderm underneath (Dupin and Sommer, 2012). Induction of NCCs involves a complex gene regulatory network that works in a feed-forward manner. Signals including BMPs, WNTs, FGFs, and Notch drive the expression of the neural plate border specific transcription factors, which superimposed on the signaling ligands to induce subsequent expression of another set of neural crest specific transcription factors (Knecht and Bronner-Fraser, 2002). In chicken embryos, the expression of *Bmp4* and *Bmp7* in the non-neural ectoderm was activated and maintained by epidermal Notch (Endo et al., 2002). Addition of Bmp4 and Bmp7 induced neural crest cells in the explant culture of chicken intermediate neural plate in the absence of non-neural ectoderm in serum-containing nutrient rich media, but not in serum-free media (Liem et al., 1995; García-Castro et al., 2002). Therefore, diffusive Bmp gradient in the ectoderm and the expression of Bmp antagonists (Noggin, Cerberus, and Chordin, etc.) in the neural plate are precisely controlled to ensure proper levels of Bmps for the induction of the neural crest (Wilson et al., 1997; Marchant et al., 1998; Tribulo et al., 2003; Sauka-Spengler and Bronner-Fraser, 2008). Elevated Bmp signaling during NCC delamination and migration has also been demonstrated in chick embryos in recent research (Piacentino et al., 2021; Rekler and Kalcheim, 2022). Likewise, canonical Wnt signaling has been well-characterized in neural crest induction, migration, and EMT in multiple vertebrates, and has been thoroughly addressed in the review by Ji et al. (2019). Wnt signaling was shown to be necessary and sufficient to induce neural crest cells in multiple organisms both *in vivo* and *in vitro*, including in *Xenopus* embryo, in chick neuroepithelium explants, as well as in human embryonic cell culture (Jones and Trainor, 2004; Devotta et al., 2018; Gomez et al., 2019). In *Xenopus* embryos, *Fgfs* were strongly expressed in the paraxial mesoderm and was sufficient to induce the expression of a panel of neural crest markers in the ectoderm independent of Wnt, as demonstrated by injection of *Fgf* expression vector *in vivo* as well as in explants (Monsoro-Burq et al., 2003). In addition, Fgf signaling from the paraxial mesoderm was also shown in chick embryo to be necessary to prevent premature specification of the caudal NCC, and thereby, prevent subsequent EMT and migration (Martínez-Morales et al., 2011; Rekler and Kalcheim, 2022).

## 4 Epithelial-mesenchymal interaction in regulating facial primordial growth

Accumulating evidence from experimental data using various animal models suggested an instructive role of epithelial signals in regulating the mesenchymal proliferation during facial primordial outgrowth. A region called frontonasal ectodermal zone (FEZ) in the frontonasal epithelium, specified by the dorsal-ventral molecular boundary of the expression of Fgf8/Shh, was first defined in stage 20 avian embryo and served as the signaling center to regulate the growth and patterning of facial primordia (Hu et al., 2003). A conserved molecular pattern of dorsal-ventral Fgf8/Shh expression was also found in mouse FEZ, which was presented as two separate signaling domains with discontinuous Shh expression on the left and right ventral side of the mouse medial nasal processes (Hu and Marcucio, 2009b; a). The bilateral pattern of Shh expression is also presented in human embryos in this region, indicating the potential fundamental differences in patterning information that distinguish mammalian faces (Odent et al., 1999). Hedgehog signaling is essential for craniofacial development, and among the three hedgehog enantiomers possessed by vertebrates, Shh has the broadest function in facial development (Abramyan, 2019). *Shh* is expressed in the facial primitive epithelium at approximately E11 in mice, and prior to its expression, facial mesenchymal cells have received Shh ligands, which possibly from the ventral neural tube epithelium, to activate the Shh signaling pathway (Kurosaka, 2015). In mouse embryos at stages between E9.5-E11.5, Fgf family members are also expressed in the facial primordium, where the ligands Fgf8, Fgf9, and Fgf10 are expressed in the facial ectoderm while Fgfr1 and Fgfr2 receptors are expressed in both facial ectoderm and mesenchyme (Bachler and Neubüser, 2001).

Using chick models, studies of ectopic transplantation of the FEZ showed the ectopic expression of the downstream genes in the facial mesenchyme and induced the subsequent alterations in patterning of morphogenesis (Hu et al., 2003). Shh induced expression of targets downstream of the Shh pathway (Ptc1 and Gli1) in the neighboring mesenchyme, while the expression of downstream targets of Fgf, Msx1 and AP2, was upregulated in the mesenchyme underneath the Fgf8-positive dorsal ectoderm even when Fgf signaling started fading at stage 25 (Hu and Helms, 1999; Hu et al., 2003). Shh can also regulate mesenchymal proliferation by modulating the forkhead box (Fox) genes (*Foxf1/2*), and both excessive and inadequate Shh signaling can lead to abnormal facial development (Hu and Helms, 1999; Jeong et al., 2004; Everson et al., 2017).

It is worth noting that the establishment of the molecular pattern of Fgf8/Shh in FEZ also relies on the regulation of other signaling pathways. For instance, eliminating canonical Wnt/β-catenin signaling in mouse facial ectoderm dramatically abolished *Fgf8* expression while induced a ventrally expanded continuous medio-lateral expression zone of *Shh* and resulted in a deformity characterized by a narrow and pointed face, resembling that was observed in avian (Reid et al., 2011). On the contrary, sustained Wnt/β-catenin signaling led to upregulated and expanded *Fgf8* expression in the entire facial ectoderm with restricted *Shh* expression in the most lateral region of FEZ, with no distinguishable craniofacial structures developed by E12.5 (Reid et al., 2011; Wang et al., 2011b). On the other hand, mesenchymal signaling also affect the molecular pattern in FEZ (Foppiano et al., 2007; Reid et al., 2011; Hu et al., 2015). Ectopic expression of *Shh* in the forebrain mesenchyme in chick embryos before facial development led to a murine-like Shh expression pattern in FEZ and a broader and shorter upper jaw resembling mouse embryo at later developmental stages (Hu and Marcucio, 2009a). In *Wnt1Cre*; *Ptch1* ^*c/c*^ mutant mouse embryos, downregulation of the expression of Ptch1, the major negative regulator/receptor of Shh, in the NCC-derived mesenchyme, resulted in aberrant upregulation of Shh signaling in the facial mesenchyme and loss-of-expression of *Fgf8* in the frontonasal epithelium (Metzis et al., 2013).

The Bmp signaling pathway regulates a series of cell proliferation, apoptosis, and differentiation processes (Wan and Cao, 2005). Upon ligand binding, Bmps activate type I and type II receptor heterodimers and in turn activate transcriptional coactivators Smads, which lead to downstream target gene expression (Wang et al., 2014). Both Bmps and Bmp antagonists have been found expressed in the epithelium as well as in the mesenchyme of facial prominences (Trousse et al., 2001). *Bmp2* and *Bmp4* are expressed in a dynamic pattern in the chick embryo facial blastoderm. *Bmp4* first expressed at HH16-17 in the facial ectoderm, and then sequentially in the mesenchyme of LnP, MnP, MxP and MdP while *Bmp2* expressed in both epithelium and mesenchyme of the facial primordium (Francis-West et al., 1994). Implantation of Noggin-soaked beads in the FnP mesenchyme at HH15/16 or infection of neural crest cells at HH10 with a replication-competent retrovirus encoding the Bmp antagonist Noggin (RCAS-Noggin) abolished Shh expression in the ventral domains of FEZ (Foppiano et al., 2007; Hu et al., 2015). Ectodermal Shh also affects the expression of Bmps in the mesenchyme, and activation of the Shh signaling pathway in FnP increases the expression of *Bmp2*, *Bmp4*, and *Bmp7* in its mesenchyme (Hu et al., 2015). Mutual antagonistical regulation was also observed between Bmp and Fgf8 signaling (Shigetani et al., 2000). *Tp63* encodes transcription factors that belong to p53 family and regulate the transcription of members of multiple signaling pathways, including Notch, Wnt, Tgf-β and Bmps (Yang et al., 2006). *Tp63* is widely expressed in the developing facial epithelium and loss-of-function in *Tp63* has been found to cause developmental malformations including OFCs in both human and mice (Thomason et al., 2008). Elevated *Bmp4* expression was shown in the epithelium of caudal LnP and MxP in *Tp63* null mice from E10.5 to E11.5, whereas the expression of *Fgf8* was absent in the overlapping region (Thomason et al., 2008). *Tp63* ^*−/−*^ mice displayed fully penetrating bilateral cleft lips due to the aberrant signaling of Bmp4 and Fgf8, which was the major positive regulator of the mesenchymal cell proliferation (Thomason et al., 2008). Correlations between the expression of the homeobox genes, *Msx1* and *Msx2*, as well as the expression of the T-box transcription factor, *Tbx22*, in the developing facial primordia and Bmp signaling have been demonstrated in both chick and mouse embryos (Barlow and Francis-West, 1997; Jiang et al., 2006; Higashihori et al., 2010). Implantation of the antagonistic Noggin beads in FnP mesenchyme before lip fusion (HH22) downregulated *Msx1/Msx2* expression in the epithelium and mesenchyme while strongly induced the mesenchymal expression of *Tbx22*, resulted in significantly inhibited mesenchymal proliferation (Ashique et al., 2002; Higashihori et al., 2010). *Tbx22* expression in facial mesenchyme was also induced by ectodermal Fgf8 and was likely to act upstream of Msx, given that gain-of-function of *Tbx22* reduced the expression of *Msx2* and *Dlx5* and resulted in craniofacial malformations including OFC (Fuchs et al., 2010; Higashihori et al., 2010). Enhanced Bmp signaling in the mesenchyme induced apoptosis of epithelial and mesenchymal cells and inhibited the expression of *Fgf8* and *Shh* in the epithelium (Ashique et al., 2002). Collectively, a role for Bmp signaling in promoting mesenchymal proliferation was supported by most of the experimental data while its function in facial ectoderm was still unclear.

Both expression analysis and association studies indicate that Wnt signaling molecules including ligands, receptors/co-receptors and downstream effectors are involved in crosstalk with other morphogenic pathways and play crucial roles in facial development (Chiquet et al., 2008; Mostowska et al., 2012; Fontoura et al., 2015; Lu et al., 2015). Mutations in genes encoding a wide range of Wnt signaling components, including both β-catenin-dependent canonical and β-catenin-independent non-canonical pathways, have been revealed in association with OFC in humans and animal models (Reynolds et al., 2019). Precisely regulated Wnt signaling is essential for the maintenance of the proper gene expression pattern in facial ectoderm as well as the mesenchymal proliferation, demonstrated by the resultant craniofacial defects of both ectodermal conditional loss-of-function (LOF) and gain-of-function (GOF) mutations of β-catenin in mice (Reid et al., 2011; Wang et al., 2011b). The expression of multiple Fgf ligands (Fgf8, Fgf17, Fgf3) was downregulated in the *β-catenin*-LOF mice while the expression of Fgf8 was expanded and upregulated in the facial ectoderm in *β-catenin*-GOF mice (Reid et al., 2011; Wang et al., 2011b). Altered expression pattern of Shh was also observed in these mice, indicating the ectodermal Wnt signaling was a player in establishing the molecular pattern in FEZ. In mouse embryos, the expression of Wnt3 and Wnt9b, both activate β-catenin mediated canonical pathway, was detected in the distal regions of the surface ectoderm of FnP and 1^st^ branchial arch derived structures as early as E9.5 (Lan et al., 2006). However, unlike *Wnt9b*, high expression of which was detected in the pre-fusion ectoderm and persistent in the epithelial seam after fusion at E11.5, the expression of *Wnt3a* was absent from the epithelial contacts in the pre-fusion zone. Loss-of-function mutations in *Wnt3* cause craniofacial defects in human and early embryonic death in mice (Liu et al., 1999). No coding mutations in *Wnt9b* has been reported in human while *Wnt9b* null mice develop incomplete penetrance of bilateral cleft lip (Jin et al., 2012). Wnt9b/β-catenin induces mesenchymal cell proliferation by regulating the expression of *Fgf8*, *Fgf10* and *Fgf17* in the ectoderm (Jin et al., 2012). Another study showed that Msx1/Msx2 were also downstream targets of the Wnt/β-catenin signaling pathway during lip formation and fusion and their expression were downregulated in the facial mesenchyme of *Wnt9b* ^*−/−*^ mice (Song et al., 2009; Jin et al., 2012). Interestingly, although the expression of Bmp4 remained unaffected in either *Wnt9b* ^*−/−*^ or *β-catenin*-LOF mouse embryos, tissue specific knockout of *Gpr177* (also known as *Wntless*, responsible for Wnt sorting/secretion) in the facial ectoderm (*Foxg1-Cre; Gpr177*) from E9.5 reduced Bmp4 expression in the facial epithelium and mesenchyme, indicating a role for non-canonical Wnt signaling in regulating Bmp4 expression (Reid et al., 2011; Wang et al., 2011b; Jin et al., 2012; Zhu et al., 2016).

**FIGURE 1:**
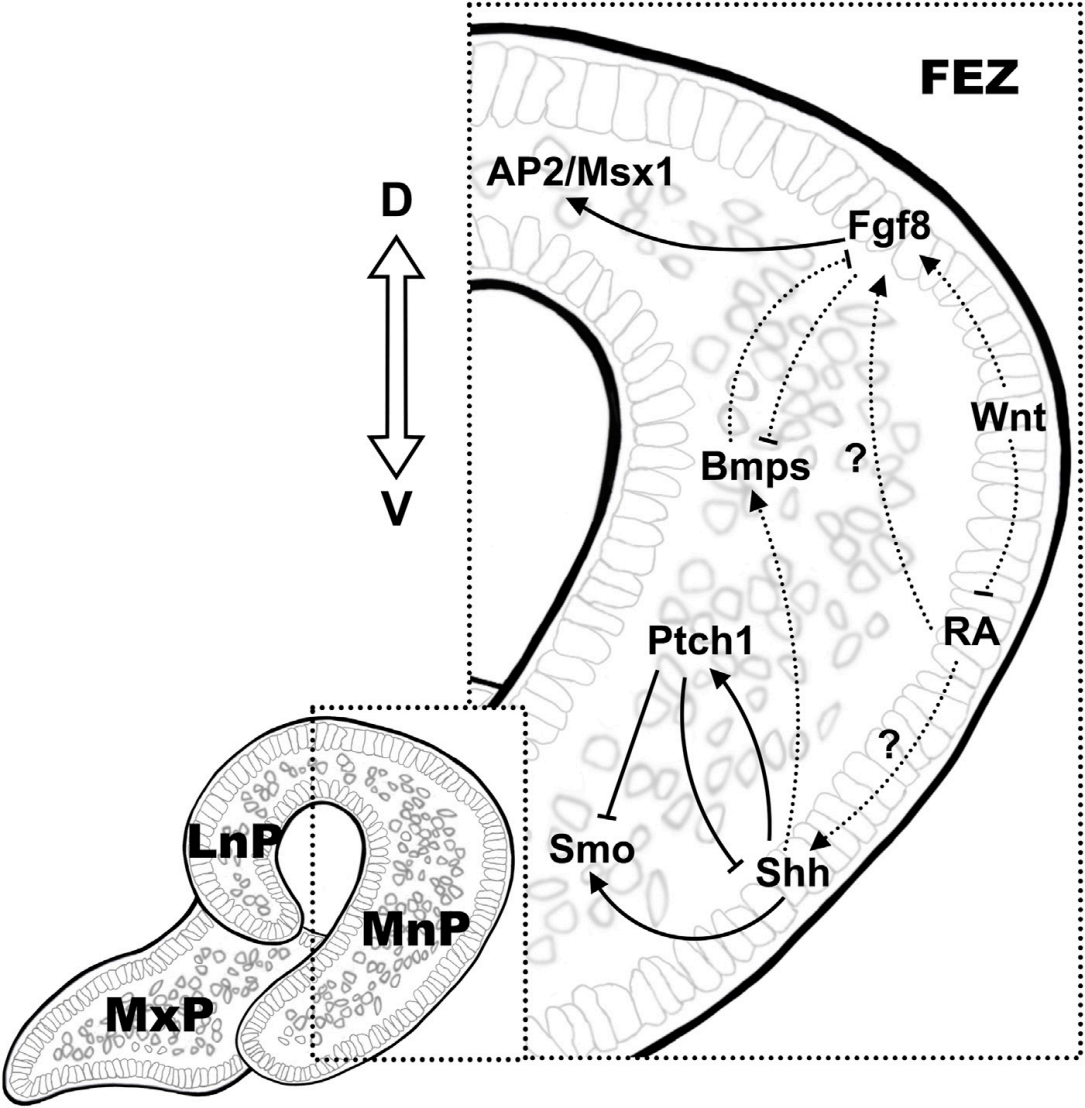
Signaling mechanisms in the establishing the molecular pattern in FEZ. The dorsal-ventral expression of Fgf8/Shh is conserved in the epithelium in FEZ, which is the signaling center in regulating mesenchymal proliferation during early facial development. Crosstalk between pathways is illustrated using solid lines and dashed lines, indicating direct and indirect regulations, respectively.

Retinoid acid, one of the metabolites of vitamin A processed by sequential enzymatic reactions involving retinol dehydrogenases and aldehyde dehydrogenases, is another signaling molecule produced in FnP ectoderm (Duester, 2000). Depends on the duration and time of commencement of induction, both excessive and deficient RA signaling lead to craniofacial defects (Emmanouil-Nikoloussi et al., 2000; Dupé and Pellerin, 2009). *Aldh6* is expressed in a restricted region in ventral epithelium of presumable FnP in chick embryos as early as at HH10-HH12 (Schneider et al., 2001). Expression of retinoid receptors including *RARα*, *RXRβ*, and *RXRγ* can be detected in the adjacent mesenchymal neural crest cells in the facial primordium (Rowe et al., 1992; Rowe and Brickell, 1995; Hoover and Glover, 1998). Transiently inhibition of the mesenchymal retinoid receptors by implanting RAR/RXR antagonists-soaked beads in the rostral head at HH10 resulted in loss of the expression of *Fgf8*/*Shh* in FnP epithelium, which in turn, led to reduced mesenchymal proliferation and increased apoptosis in the FnP mesenchyme (Schneider et al., 2001). On the other hand, RA signaling was subjected to negative regulation by Wnt, shown by the expanded expression of *Raldh3* in the pre-fusion epithelium of the upper lip/nasal primordia in the *Lrp6* ^*−/−*^ mice that displayed inactivated Wnt signaling and full penetrance of cleft lip/palate (Song et al., 2009) (Figure 1).

## 5 Epithelial-mesenchymal interaction in regulating palatal development

The Fgf-Shh signaling network involved in epithelial-mesenchymal interactions has also been shown to regulated palate development in mouse (Rice et al., 2004; Lan and Jiang, 2009). Embryonic palatogenesis displayed anteroposterior polarity with anterior bony hard palate and posterior muscular soft palate as well as oronasal polarity before fusion (Bush and Jiang, 2012). During palatal development (E12-14), the Fgf10 ligand is preferentially expressed in oral region of the anterior palatal mesenchyme adjacent to the epithelium while the receptor Fgfr2b is expressed in the palatal epithelium (Rice et al., 2004; Teshima et al., 2016). Both *Fgfr2b* ^*−/−*^ and *Fgf10* ^*−/−*^ mice have cleft palate and reduced proliferation of palatal shelf cells (Rice et al., 2004). The expression of Shh is restricted to the palatal epithelium on the oral side from E11.5 to E15.5, with the receptor Ptch1 and downstream transcription factor, Gli1, express in both palatal epithelium and mesenchyme (Ingham and McMahon, 2001; Lan and Jiang, 2009; Economou et al., 2012). Upon ligand binding, Ptch1 releases Smo from inhibition, which in turn activates GliFL and promotes target gene expression (Cohen et al., 2015). Epithelial Shh signal also regulated the palatal mesenchymal cell proliferation by modulating the expression of cycle D1/2 (Ccnd1/2), which was essential for palatal shelf growth (Lan and Jiang, 2009). Fgf10-Fgfr2b maintains *Shh* expression in palatal epithelium and *vice versa*, disrupted Shh signaling resulted in downregulation of *Fgf10* in in palatal bone mesenchyme (Rice et al., 2004; Lan and Jiang, 2009). *Odd-jump-related 2* (*Osr2*), homolog of *Drosophila Odd-skipped*, encoding a zinc finger protein of transcription factor that expressed in mouse embryos as early as E9.25 in the mesonephros, regulates pattern formation in a wide range of embryonic structures (Lan et al., 2001). *Osr2* was highly expressed in the mesenchyme of palate shelves at E12.5 and the expression were decreased by E14.5 (Lan et al., 2001). *Osr2* ^−/−^ mouse embryos displayed cleft palate and reduced Fgf10 expression in the palatal mesenchyme (Zhou et al., 2013). In *Osr2-Cre*; *Smo* ^*c/c*^ mouse embryos *in vivo* as well as in mouse palate shelf cultures *in vitro*, dysregulation in Shh-Smo signaling also altered *Bmp2*/*Bmp4* expression in the underlying mesenchyme in the anterior palate, upregulated *Bmp2* expression induced by exogenous Shh while reduced *Bmp2* expression and increased *Bmp4* expression in *Osr2-Cre*; *Smo* ^*c/c*^ mouse palate shelves (Lan and Jiang, 2009).

Another member of the Fgf family, Fgf7, is nevertheless expressed in the nasal region of the palatal mesenchyme and is regulated by Dlx5, which displayed overlapping expression pattern (Han et al., 2009). The expression of Fgf7 in the oral region of palate mesenchyme was repressed by Shh. Loss-of-function of *Dlx5* resulted in downregulation of Fgf7 and the expansion of Shh to the nasal side of palatal epithelium in *Dlx* ^*−/−*^ mice that displayed cleft palates with reduced proliferation of mesenchymal cells (Han et al., 2009). However, excessive Shh signaling in in palatal epithelium also caused cleft palate (Cobourne et al., 2009). Therefore, it was observed that the palatal fusion defects in palato-mesenchymal proliferation-deficient (*Msx* ^*−/−*^) mice were rescued by the expanded Shh expression in *Msx1* ^*−/−*^ *Dlx5* ^*−/−*^ double knockout mice (Han et al., 2009). Inhibition of Shh expression by expression of Fgf18 and Fgf8 in palate mesenchyme has also been shown in mice. The expression of Shh in palate epithelium was significantly downregulated in conditional knockout mice of *Foxf1/2*, which displayed ectopic overexpression of Fgf18 in the oral side of palatal mesenchyme while addition of exogenous Fgf18 protein to cultured palatal explants suppressed Shh expression in the palatal epithelium (Xu et al., 2016). Activation of ectopic Fgf8 expression in the palatal mesenchyme in *Osr2-CreKI*; *Rosa26R-Fgf8* mice resulted in dramatically increased proliferation of posterior palatal mesenchymal cells whereas decreased epithelial cell proliferation due to downregulated Shh expression (Wu et al., 2015). Further investigations suggested the inhibition of Shh expression in the palate epithelium by Fgf8 was secondary, likely to be the outcome of disrupted Shh-Fgf10 feedback loop (Wu et al., 2015). In addition, ectopic activation of *Fgf8* also suppressed the expression of Dlx5 and Fgf7 (Wu et al., 2015). Therefore, the altered expression of Fgf8 in mouse embryonic palate may affect cell proliferation via a combined mechanisms involving multiple EMI pathways.

Bmp signaling is critical in the regulation of palatal growth and mesenchymal condensation/ossification (Parada and Chai, 2012). In mouse palatogenesis, the expression of Bmp4 was observed in the palatal epithelium and mesenchyme at E12.5 and was restricted to the anterior palatal mesenchyme at E13.5, while the expression of Bmp2 was observed in the epithelium and mesenchyme of the anterior region of the palatal shelf (Zhang et al., 2002). Inactivation of Shh signaling in the palatal mesenchyme by tissue specific knockout of *Smo* in the palatal mesenchyme (Osr2-IresCre; Smoc^/c^) led to overexpression of *Bmp4* and *Msx1* while led to downregulation of *Bmp2* (Lan and Jiang, 2009). However, proper Bmp4 signaling is also essential for maintaining Shh expression in the medial edge epithelium, evidenced by the restored *Shh* and *Bmp2* expression as well as the cell proliferation in *Msx* ^*−/−*^ mice by ectopic *Bmp4* expression (Zhang et al., 2002). Given that Bmp4 was shown to act downstream of Msx1, the induced Msx1 expression in palatal explant cultures by implantation of Bmp4-soaked beads might be the consequence of the inhibited Shh expression due to the feedback regulation between Shh and Bmp4 (Chen et al., 1996; Zhang et al., 2002; Hilliard et al., 2005). The Bmp antagonist Noggin is also expressed in the palatal epithelium (He et al., 2010). *Noggin* ^*−/−*^ mouse embryos displayed defective proliferation and excessive apoptosis in the mesenchyme of palatal shelves. Reduced Smad1/5/8 phosphorylation in the anterior palatal mesenchyme and upregulation of Smad1/5/8 along the A-P axis in the posterior palatal mesenchyme have been demonstrated in these mouse embryos, whereas Bmp2 is reduced in the anterior palatal mesenchyme and ectopically expressed in the posterior palatal oral side of the epithelium (He et al., 2010). The altered expression of Bmp2 along the A-P axis in *Noggin* ^*−/−*^ mutants might be attributed in part to the changes in the levels of Smad1/5/8 phosphorylation in the palatal mesenchyme. Conditional knockout of the receptor *Bmpr1a* in the palatal mesenchyme and epithelium in mice (*Nestin-Cre*; *Bmpr1a* ^*f/-*^) results in cleft lip and cleft palate (Liu et al., 2005). Further knockout studies involving tissue specific inactivation of *Bmpr1a* in mouse palatal mesenchyme (*Osr2-IresCre*; *Bmpr1a* ^*f/f*^) showed that the expression of Shh and Msx1/Fgf10 was downregulated in the anterior palatal epithelium and mesenchyme, respectively, while the expression of Bmp4 and Bmp2 was increased in compensation in the palatal mesenchyme (Baek et al., 2011). These mice displayed cleft in anterior secondary palate due to reduced cell proliferation. However, deletion of *Bmpr1a* expression in the palatal epithelium only (*K14-Cre*; *Bmpr1a* ^*f/-*^) resulted in normal palatal development (Andl et al., 2004).

The expression of Gpr177 is found in both palatal epithelium and mesenchyme and promotes Wnt ligands secretion (Bänziger et al., 2006; Liu et al., 2015). Elimination of Wnt signaling by conditional knockout of *Gpr177* in NCC-derived mesenchyme (*Gpr177*; *Wnt1-Cre*) resulted in abnormal cell proliferation and increased cell death in the palatal shelf, accompanied with upregulated expression of Msx1 in the anterior palatal mesenchyme and increased palatal epithelial Shh expression (Liu et al., 2015). Similar to its obligatory roles in establishing Fgf/Shh expression pattern in FEZ, activated Wnt signaling and high expression of β-catenin was also observed in the palatal rugae epithelium, which served as the Shh signaling center in palatal development (Lin et al., 2011). Conditional inactivation of β-catenin in the palatal epithelium at the beginning of palatal development at E12.5 in mouse embryos ablated Shh expression in the palatal rugae and consequently, disrupted the A-P expansion of the palatal rugae and palatine bone development (Lin et al., 2011). Wnt5a was expressed in gradience along the A-P axis in the mesenchyme during mouse palatogenesis (He et al., 2008). Downregulated Bmp4 expression in the mesenchyme and Shh in MEE of the anterior palate whereas ectopic Bmp4 expression in the mesenchyme and Shh expression in the nasal side epithelium of the posterior palate were observed in E13.5 *Wnt5a* ^*−/−*^ mouse embryos, which displayed complete cleft palate (He et al., 2008). The expression of Shh receptor, Ptch1, and the downstream target of Bmp4, Msx1, changed accordingly in the developing palate of *Wnt5a* ^*−/−*^ mouse embryos (He et al., 2008). These observations were in line with the epistatic effect of Wnt signaling in directing the establishment of the molecular pattern during upper lip formation. Null mutation in the Wnt5a receptor, *Ror2*, resulted in similar alterations in the gene expression in mouse embryonic palate and overlapping phenotype, indicating a non-canonical Wnt pathway in the regulation of palatogenesis.

Enzymes of cytochrome P450 subfamily 26 (CYP26) play dominant roles in RA degradation and display distinctive expression pattern in embryonic palate, with restricted expression of *Cyp26a1* in the epithelium and *Cyp26b1* in the mesenchyme (White et al., 1997; Okano et al., 2012). *Cyp26b1* ^*−/−*^ mouse embryos suffered from cleft palate, displaying downregulated expression of *Fgf10* and *Bmp2* in the palatal mesenchyme and downregulated *Tbx1* in the palatal epithelium, respectively (Okano et al., 2012). Both the expression of *Cyp26a1* and *Cyp26b1* in the palatal shelf was reduced in *ShhCreER* ^*T2*^ */Shh* ^*f*^ mutant mice, suggested an epistatic inhibitory effect of epithelial Shh signaling in regulating mesenchymal and epithelial RA signaling (El Shahawy et al., 2019). Antagonistic interaction was also found between RA signaling and Tgfβ pathway in regulating palatogenesis. Exogenous RA induced apoptosis in palatal mesenchymal cells and prevented palatal shelf fusion by inhibiting the Tgfβ-Smad pathway in the MES and palatal mesenchyme, and *vice versa* (Yu et al., 2005; Yu and Xing, 2006; Wang et al., 2011a; Liu et al., 2014).

In addition, these aforementioned signaling pathways are integrated to regulate the composition of the extracellular matrix, which served as the fundamental infrastructure for cell adhesion to guide the directional growth and elevation of embryonic palate (Wang et al., 2020). For instance, Collagens (Col), the major component of the extracellular matrix (ECM), are expressed extensively in the palatal mesenchyme before and after the elevation of the PS (Silver et al., 1981). The forkhead transcription factor, Foxf2, induced by ectodermal Shh and expressed in the cranial neural crest cells in MnP/MdP, activate canonical Tgfβ signaling in the palatal mesenchyme (Nik et al., 2016; Xu et al., 2016). The reduced expression of Tgf-β2 resulted in mesenchymal hypoplasia together with reduced production of Col I and several other extracellular proteins in *Foxf2* ^*−/−*^ mouse embryos that displayed cleft secondary palate (Nik et al., 2016). In contrast, the other two Tgfβ ligands, Tgfβ1 and Tgf-β3 are expressed in the palatal epithelium including MEE (Fitzpatrick et al., 1990). Tgf-β1 induced the proliferation and Col I and III synthesis in cultured palatal mesenchymal cells *in vitro* (Li et al., 2012). Downregulation of Tgf-β3 reduced Shh expression throughout E12.5-E15.5 in palate rugae and resulted in the failure of palate fusion without significant reduction in palatal growth (Sasaki et al., 2007).

**FIGURE 2:**
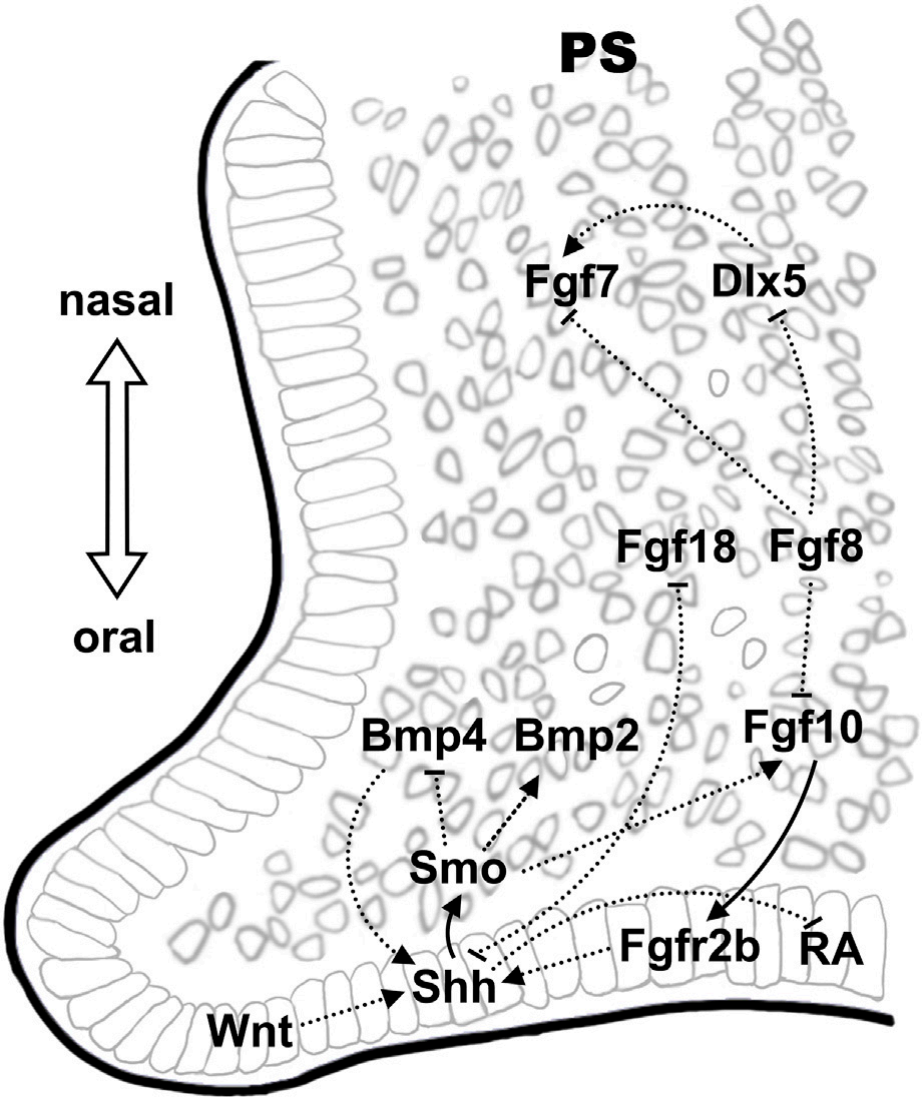
Epithelial-mesenchymal signaling interactions during palate development. The Shh-Fgf signaling network, which display oronasal polarity, is also found critical in palatogenesis. Wnt signaling in the epithelium on the oral side demonstrate an epistatic effect in regulating Shh expression. Crosstalk between pathways is illustrated using solid lines and dashed lines, indicating direct and indirect regulations, respectively.

Mutual regulatory mechanisms are also commonly recognized between the signaling pathways and the transcription factors. The basic helix-loop-helix (bHLH) transcription factors dHAND/HAND2 is expressed in cephalic neural crest cells as early as at E9.5 just after migration, as demonstrated using the dHAND-lacZ transgenic mice (Massari and Murre, 2000; Ruest et al., 2003; Barbosa et al., 2007). At later developmental stages, expression of Hand2 is found in the anterior palatal mesenchyme and MEE and the expression was ablated in *Osr2-Cre; pMes-Nog* mice that had attenuated Bmp signaling in the mesenchyme (Xiong et al., 2009). Inactivation of *Hand2* in the palatal mesenchyme had no effect on palatal development while elimination of *Hand2* in the palatal epithelium led to cleft palate resulted from premature death of periderm cells in the MEE and reduced mesenchymal proliferation via modulating Shh expression (Morikawa et al., 2007; Xiong et al., 2009). Early expression of the paired-box transcription factor, Pax9, in the palate region, became detectable at E12.5 in the mesenchyme in a posterior-anterior gradient pattern as well as in the posterior epithelium (Zhou et al., 2013). This was followed by a gradual decrease of expression in the mesenchyme, accompanied by intensely confined expression in the MEE during palate fusion (E14.5-E15.5) (Sasaki et al., 2007). The mutant mice exhibit cleft palate and die shortly after birth (Kist et al., 2007). The expression of *Pax9* was regulated by epithelial Fgf signaling and exogenous *Fgf8* induced *Pax9* expression in the posterior palatal mesenchyme (Hilliard et al., 2005). The expression of *Shh* in the palatal rugae as well as the expression of *Bmp4*, *Fgf10*, *Msx1* and *Osr2* in the palatal mesenchyme were significantly downregulated in *Pax9* null mice, suggesting Pax9 as a core player in converting the signaling cues to mediate the communication between epithelium and mesenchyme (Zhou et al., 2013) (Figure 2).

## 6 Epithelial-mesenchymal interaction in the processes of lip and palate fusion

The growth and expansion of the facial prominences eventually lead to their fusion in the midline, which is a process involving epithelial seam formation followed by EMT and/or epithelial regression (Gaare and Langman, 1977; Sun et al., 2000). Fusion is a sequential and directional process that has species differences, with fusion initiated between MnP and LnP in posterior-anterior direction followed by the fusion between MnP and MxP in mice (Jiang et al., 2006). *Wnt9b* is expressed in the epithelial seam between the fused MnP and LnP (Lan et al., 2006). Meeting of MnP, LnP and MxP create a three-way seam called lambdoidal junction, where Wnt-p63-Irf6 are intensively expressed to maintain apoptosis in epithelial suture cells (Ferretti et al., 2011). The Wnt-p63-Irf6 regulatory module was disrupted in *Ptch1*wiggable mice embryos that was shown to have enhanced Shh signaling in the mesenchyme (Kurosaka et al., 2014). Further investigations showed that multiple canonical Wnt inhibitors’, such as *Vax1*, *Sfrps* and *Frzb*, were upregulated while the expression of the transcription factor of *Irf6* promotor, *Tfap2a*, was downregulated in the mesenchyme of facial processes of *Ptch1* wiggable mice (Kurosaka et al., 2014).

The periderm is a barrier that prevents any unwanted adhesion of the palatal shelf to the surrounding tissues and the failure of periosteum differentiation leads to Cleft Lip and Palate (CLP) (Hammond et al., 2019). Expression of *Irf6* and its downstream target, the receptor-interacting protein kinase 4 (*RIPK4*), was observed throughout mouse palatogenesis in the palatal epithelium and was important for peridermal formation (De Groote et al., 2015; Kousa and Schutte, 2016). Mutations in these genes resulted in anomalies including cleft palate as well as abnormal adhesion of the palatal shelves to the mandible and/or tongue due to impaired peridermal differentiation and ectopic apical E-cadherin distribution, respectively (Ingraham et al., 2006; De Groote et al., 2015). Jag2, one of the surface ligands of the receptors of Notch family, mediates the process of differentiation in the oral periderm and the mutant mice displayed CLP with palate-tongue fusion (Shawber et al., 1996). In mice, high expression of Jag2 was observed in the tongue, mandibular and maxillary epithelia from E12.5 to E14.5, with its receptor, Notch1 expressed in overlapping regions with a time delay at E13.5 (Casey et al., 2006). Downregulated *Jag2* expression in the oral epithelia was observed in *Fgf10* ^*−/−*^ mutant mice that displayed abnormal palate-tongue fusion and likewise, although *Fgf10* expression was unaltered in the mesenchyme, reduced *Fgfr2b* expression in the oral epithelium was observed in *Jag2* mutant mice, indicating that the Fgf signaling in the mesenchyme might regulate the epithelial differentiation by a feedback regulation of Jag2-Notch signaling (Alappat et al., 2005). The transcription factor Tbx1 that is expressed in epithelial cells of early facial processes has also been found expressed in palatal epithelium in mice from E12.5 to E15.5 (Funato et al., 2012). The results of expression analysis suggested altered gene expression of *Fgf10*, *Bmp4* and *Pax9*, etc., in the palatal mesenchyme of *Tbx1* ^*−/−*^ mice that showed cleft palate with abnormal epithelial adhesions between the palatal shelf and the mandible (Goudy et al., 2010; Funato et al., 2012). These findings may partly explain the defective mesenchymal proliferation as well as impaired epithelium differentiation observed during palatogenesis in *Tbx1*-null mice (Funato et al., 2012; Zoupa et al., 2018).

**FIGURE 3:**
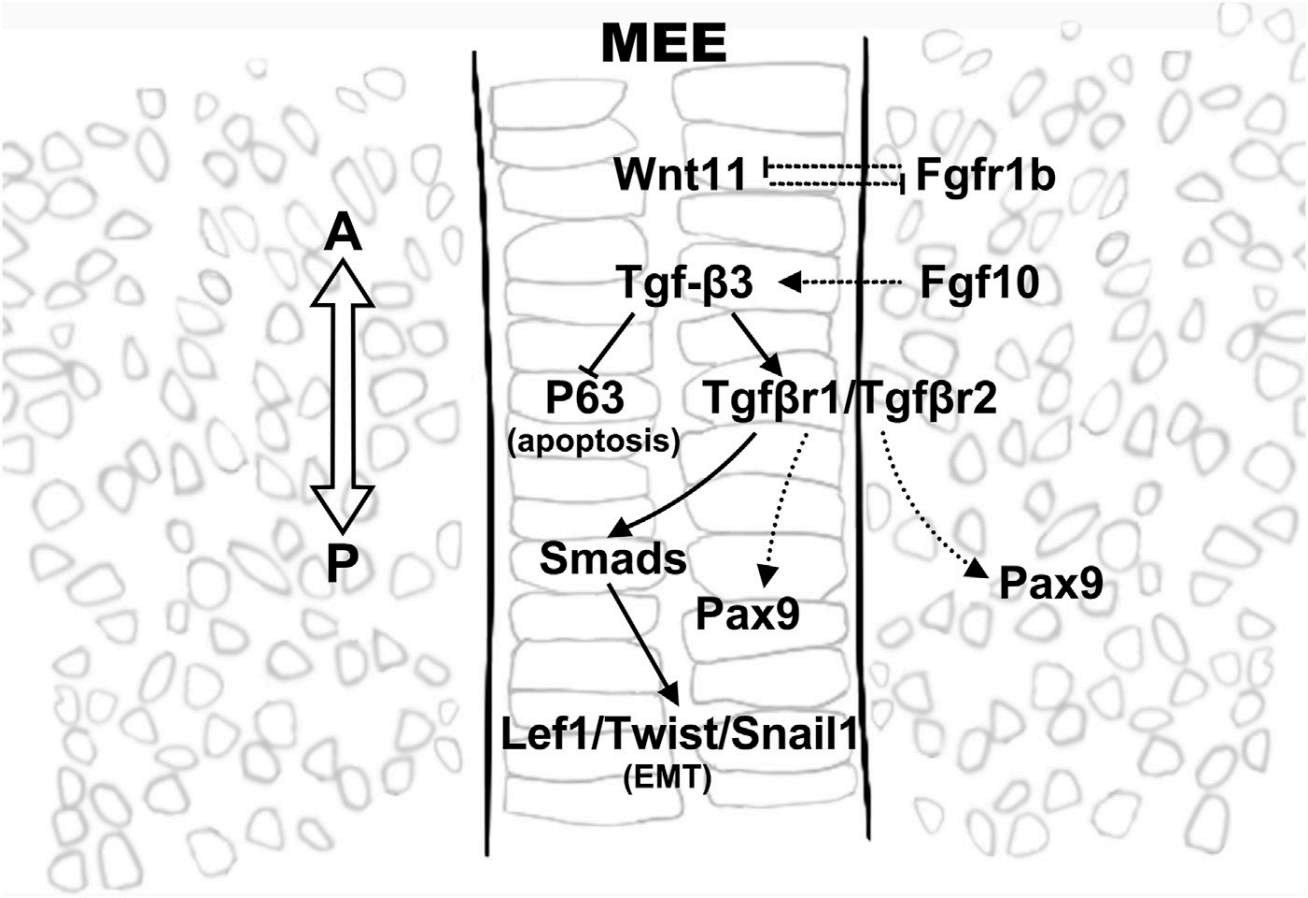
Tgf-β3 is a key player in regulating epithelial fate in MEE. Tgf-β3 is expressed before and after palatal fusion in MEE, where it plays a key role in determining the epithelial cell fate by regulating the expression of a wide range of downstream genes that involved in apoptosis as well as in EMT. Crosstalk between pathways is illustrated using solid lines and dashed lines, indicating direct and indirect regulations, respectively.

The growth and elevation of palatal shelves lead to their fusion in the midline above the tongue. The epithelial cells in the medial edge epithelia are subjected to three different fates, programmed cell death, migration or EMT, fail in which resulted in submucous cleft (Jin and Ding, 2006). *Wnt11* is strongly expressed in the MEE region and *Fgfr1b* is expressed in the palatal mesenchyme (Lee et al., 2008). The mutual inhibitory regulation between Wnt11 and Fgfr1b was shown to be critical for the apoptosis during fusion in cultured palatal shelves *in vitro* (Lee et al., 2008). Initial palatal shelf contact is mediated by chondroitin sulfate proteoglycans expressed on filamentous pseudopods that formed on the apical surface of periderm cells, followed by migration of periderm cells out of the MES (Taya et al., 1999; Martínez-Alvarez et al., 2000; Cuervo and Covarrubias, 2004; Vaziri Sani et al., 2010; Richardson et al., 2017). *Tgf-β3* is expressed in MEE and knockdown of *Tgf-β3* or epithelial-specific deletion of *Tgfbr1* or *Tgfbr2* results in impaired MEE degeneration and palatal fusion (Proetzel et al., 1995; Dudas et al., 2006; Xu et al., 2006; Nakajima et al., 2018). The pattern of Tgf-β3 expression was regulated by Fgf10 in the palatal mesenchyme and the expression of Tgf-β3 in the mid-posterior palatal epithelium was expanded to the oral side in *Fgf10* ^*−/−*^ mice at stage E13.5 (Alappat et al., 2005). On the other hand, Tgf-β3 also affected the gene expression in the palatal mesenchyme. As aforementioned, Pax9 was a key modulator of a few signaling molecules in palatal epithelium. Its expression was vividly detected in the palatal mesenchyme at E12.5 and became intensified in the medial edge epithelium during fusion (from E13.5 to E14.5) (Sasaki et al., 2007). The expression of Pax9 was significantly downregulated at E13.5 and was completely absent from E14.5 to E15.5 in the palatal region in *Tgf-β3* ^*−/−*^ mice (Sasaki et al., 2007). Fail in the formation of pseudopods and chondroitin sulfate proteoglycans synthesis might account for the impaired migration of periderm cells in the MEE of the *Tgf-β3* ^*−/−*^ mice (Taya et al., 1999; Richardson et al., 2017). Tgf-β3 has also been suggested to play a role in regulating EMT via Smad-dependent and Smad-independent signaling pathways to regulate the expression of the EMT-related transcription factors Lef1, Twist, and Snail1, etc., in the epithelial cells in MEE (Yu et al., 2009). In the epithelial cells in the seam, Tgf-β3 also downregulated the expression of p63, which acted directly on the promoters of a variety of genes (Pvrl1, Irf6, Fgfr2, Tcfap2a, Pdgfa, Sfn, Grhl3 and Jag2) that were involved in the determination of the fate of the peridermal cells (Moretti et al., 2010; Thomason et al., 2010). The detailed mechanisms of Tgf-β3 in the regulation of MEE cell fate have been elaborated in excellent reviews by Akira Nakajima et al. (Yu et al., 2009; Nakajima et al., 2018; Hammond et al., 2019; Hammond and Dixon, 2022). Other examples of the epithelial-mesenchymal interaction in regulating palate fusion include mesenchymal Fgf18, which induced the expression of the transcription factor Runx1 in the fusing epithelium from E13.5 to E15.5 (Charoenchaikorn et al., 2009) (Figure 3).

## 7 Summary and perspectives

Epithelial-mesenchymal interactions are one of the fundamental essential developmental events during morphogenesis and organ formation. Mechanisms involved in mediating epithelial-mesenchymal interactions include soluble signaling molecules, direct cell-cell contacts and the components of extracellular matrix. In spite of the sharing molecular mechanisms (i.e., Fgf, hedgehog, Wnt, Tgf-β and Bmps) that govern different organ development, the ectodermal epithelium of facial prominences plays a dominant inductive role during craniofacial development while the instructive function is more likely to be ascribed to the mesenchyme in lung and kidney formation (Ribatti and Santoiemma, 2014). The integrity of facial epithelia that derived from surface ectoderm ensures the spatiotemporal regulation of mesenchymal proliferation and migration as well as subsequent fusion at ventral midline (Lan and Jiang, 2022). The best example came from the development of FEZ, a facial ectoderm domain defined by the reciprocal exclusive expression of Shh and Fgf8 that played morphogenic roles in craniofacial development, as evidenced by numerous conventional knockout and transplantation studies in both chicken and mice as well as by recent quantitative morphometric analysis (Hu et al., 2003; Xu et al., 2015). On the other hand, alternation of mesenchymal signaling actively changed the molecular pattern formation in the ectoderm (Foppiano et al., 2007; Hu and Marcucio, 2012). Compared to the remarkable progress that has been made in resolving the genetic etiology of OFCs by identifying potential causative genes using high throughput technologies, little has been achieved in the understanding of the molecular pathogenesis of these malformations (Sull et al., 2009; Saleem et al., 2019; Yan et al., 2020). Given the active interplay between the facial epithelium and the mesenchyme, effective therapeutic strategies may be developed by intervening in this process.

## Abbreviations

EMI, Epithelial-mesenchymal interaction; OFC, Orofacial cleft; SES, Socioeconomic status; FnP, Frontonasal prominence; MnP, Medial nasal prominence; LnP, Lateral nasal prominence; MxP, Maxillary prominence; MdP, Mandibular prominence; NCC, Neural crest cell; Shh, Sonic hedgehog; Fgf, Fibroblast growth factor; Bmp, Bone morphogenetic protein; Tgf-β, Transforming growth factor-β; Wnt, Wingless-type MMTV integration site; RA, Retinoic acid; EMT, Epithelial-mesenchymal transition; CNCC, Cranial neural crest cell; MEE, Medial edge epithelia; MES, Medial epithelial seam; FEZ, Frontonasal ectodermal zone; Fox, Forkhead box; LOF, Loss-of-function; GOF, Gain-of-function; Osr2, Odd-jump-related 2; Cyp26, Cytochrome P450 subfamily 26; Col, Collagen; ECM, Extracellular matrix; bHLH, basic helix-loop-helix; CLP, Cleft Lip and Palate; RIPK4, Receptor-interacting protein kinase 4.

## References

[B1] Abramyan J. (2019). Hedgehog signaling and embryonic craniofacial disorders. J. Dev. Biol. 7, 9. 10.3390/jdb7020009 31022843 PMC6631594

[B2] Alappat S. R. Zhang Z. Suzuki K. Zhang X. Liu H. Jiang R. (2005). The cellular and molecular etiology of the cleft secondary palate in Fgf10 mutant mice. Dev. Biol. 277, 102–113. 10.1016/j.ydbio.2004.09.010 15572143

[B3] Andl T. Ahn K. Kairo A. Chu E. Y. Wine-Lee L. Reddy S. T. (2004). Epithelial Bmpr1a regulates differentiation and proliferation in postnatal hair follicles and is essential for tooth development. Development 131, 2257–2268. 10.1242/dev.01125 15102710

[B4] Ashique A. M. Fu K. Richman J. M. (2002). Endogenous bone morphogenetic proteins regulate outgrowth and epithelial survival during avian lip fusion. Development 129, 4647–4660. 10.1242/dev.129.19.4647 12223420

[B5] Atukorala A. D. S. Ratnayake R. K. (2021). Cellular and molecular mechanisms in the development of a cleft lip and/or cleft palate; insights from zebrafish (*Danio rerio*). Anatomical Rec. 304, 1650–1660. 10.1002/ar.24547 33099891

[B6] Bachler M. Neubüser A. (2001). Expression of members of the Fgf family and their receptors during midfacial development. Mech. Dev. 100, 313–316. 10.1016/s0925-4773(00)00518-9 11165488

[B7] Baek J. A. Lan Y. Liu H. Maltby K. M. Mishina Y. Jiang R. (2011). Bmpr1a signaling plays critical roles in palatal shelf growth and palatal bone formation. Dev. Biol. 350, 520–531. 10.1016/j.ydbio.2010.12.028 21185278 PMC3031756

[B8] Bänziger C. Soldini D. Schütt C. Zipperlen P. Hausmann G. Basler K. (2006). Wntless, a conserved membrane protein dedicated to the secretion of Wnt proteins from signaling cells. Cell 125, 509–522. 10.1016/j.cell.2006.02.049 16678095

[B9] Barbosa A. C. Funato N. Chapman S. Mckee M. D. Richardson J. A. Olson E. N. (2007). Hand transcription factors cooperatively regulate development of the distal midline mesenchyme. Dev. Biol. 310, 154–168. 10.1016/j.ydbio.2007.07.036 17764670 PMC2270479

[B10] Barlow A. J. Francis-West P. H. (1997). Ectopic application of recombinant BMP-2 and BMP-4 can change patterning of developing chick facial primordia. Development 124, 391–398. 10.1242/dev.124.2.391 9053315

[B11] Bush J. O. Jiang R. (2012). Palatogenesis: morphogenetic and molecular mechanisms of secondary palate development. Development 139, 231–243. 10.1242/dev.067082 22186724 PMC3243091

[B12] Carlson K. J. Byron C. D. (2008). Building a better organismal model: the role of the mouse--Introduction to the symposium. Integr. Comp. Biol. 48, 321–323. 10.1093/icb/icn068 21669794

[B13] Casey L. M. Lan Y. Cho E. S. Maltby K. M. Gridley T. Jiang R. (2006). Jag2-Notch1 signaling regulates oral epithelial differentiation and palate development. Dev. Dyn. 235, 1830–1844. 10.1002/dvdy.20821 16607638 PMC3869087

[B14] Charoenchaikorn K. Yokomizo T. Rice D. P. Honjo T. Matsuzaki K. Shintaku Y. (2009). Runx1 is involved in the fusion of the primary and the secondary palatal shelves. Dev. Biol. 326, 392–402. 10.1016/j.ydbio.2008.10.018 19000669

[B15] Chen Y. Bei M. Woo I. Satokata I. Maas R. (1996). Msx1 controls inductive signaling in mammalian tooth morphogenesis. Development 122, 3035–3044. 10.1242/dev.122.10.3035 8898217

[B16] Chiquet B. T. Blanton S. H. Burt A. Ma D. Stal S. Mulliken J. B. (2008). Variation in WNT genes is associated with non-syndromic cleft lip with or without cleft palate. Hum. Mol. Genet. 17, 2212–2218. 10.1093/hmg/ddn121 18413325 PMC2852032

[B17] Clark J. Mossey P. Sharp L. Little J. (2003). Socioeconomic status and orofacial clefts in Scotland, 1989 to 1998. Cleft palate-craniofacial J. 40, 481–485. 10.1597/1545-1569_2003_040_0481_ssaoci_2.0.co_2 12943441

[B18] Cobourne M. T. Xavier G. M. Depew M. Hagan L. Sealby J. Webster Z. (2009). Sonic hedgehog signalling inhibits palatogenesis and arrests tooth development in a mouse model of the nevoid basal cell carcinoma syndrome. Dev. Biol. 331, 38–49. 10.1016/j.ydbio.2009.04.021 19394325 PMC2696601

[B19] Cohen M. Kicheva A. Ribeiro A. Blassberg R. Page K. M. Barnes C. P. (2015). Ptch1 and Gli regulate Shh signalling dynamics via multiple mechanisms. Nat. Commun. 6, 6709. 10.1038/ncomms7709 25833741 PMC4396374

[B20] Cuervo R. Covarrubias L. (2004). Death is the major fate of medial edge epithelial cells and the cause of basal lamina degradation during palatogenesis. Development 131, 15–24. 10.1242/dev.00907 14645125

[B21] De Groote P. Tran H. T. Fransen M. Tanghe G. Urwyler C. De Craene B. (2015). A novel RIPK4-IRF6 connection is required to prevent epithelial fusions characteristic for popliteal pterygium syndromes. Cell Death Differ. 22, 1012–1024. 10.1038/cdd.2014.191 25430793 PMC4423184

[B22] Devotta A. Hong C. S. Saint-Jeannet J. P. (2018). Dkk2 promotes neural crest specification by activating Wnt/β-catenin signaling in a GSK3β independent manner. Elife 7, e34404. 10.7554/eLife.34404 30035713 PMC6056231

[B23] Dudas M. Kim J. Li W. Y. Nagy A. Larsson J. Karlsson S. (2006). Epithelial and ectomesenchymal role of the type I TGF-beta receptor ALK5 during facial morphogenesis and palatal fusion. Dev. Biol. 296, 298–314. 10.1016/j.ydbio.2006.05.030 16806156 PMC1557652

[B24] Duester G. (2000). Families of retinoid dehydrogenases regulating vitamin A function: production of visual pigment and retinoic acid. Eur. J. Biochem. 267, 4315–4324. 10.1046/j.1432-1327.2000.01497.x 10880953

[B25] Dupé V. Pellerin I. (2009). Retinoic acid receptors exhibit cell-autonomous functions in cranial neural crest cells. Dev. Dyn. 238, 2701–2711. 10.1002/dvdy.22087 19777591

[B26] Dupin E. Sommer L. (2012). Neural crest progenitors and stem cells: from early development to adulthood. Dev. Biol. 366, 83–95. 10.1016/j.ydbio.2012.02.035 22425619

[B27] Economou A. D. Ohazama A. Porntaveetus T. Sharpe P. T. Kondo S. Basson M. A. (2012). Periodic stripe formation by a Turing mechanism operating at growth zones in the mammalian palate. Nat. Genet. 44, 348–351. 10.1038/ng.1090 22344222 PMC3303118

[B28] El Shahawy M. Reibring C. G. Hallberg K. Neben C. L. Marangoni P. Harfe B. D. (2019). Sonic hedgehog signaling is required for Cyp26 expression during embryonic development. Int. J. Mol. Sci. 20, 2275. 10.3390/ijms20092275 31072004 PMC6540044

[B29] Emmanouil-Nikoloussi E. N. Goret-Nicaise M. Foroglou C. H. Katsarma E. Dhem A. Dourov N. (2000). Craniofacial abnormalities induced by retinoic acid: a preliminary histological and scanning electron microscopic (SEM) study. Exp. Toxicol. Pathol. 52, 445–453. 10.1016/s0940-2993(00)80080-9 11089896

[B30] Endo Y. Osumi N. Wakamatsu Y. (2002). Bimodal functions of Notch-mediated signaling are involved in neural crest formation during avian ectoderm development. Development 129, 863–873. 10.1242/dev.129.4.863 11861470

[B31] Erickson A. G. Kameneva P. Adameyko I. (2023). “The transcriptional portraits of the neural crest at the individual cell level,” in Seminars in cell and developmental biology (Elsevier), 68–80. 10.1016/j.semcdb.2022.02.017 PMC9441473 35260294

[B32] Everson J. L. Fink D. M. Yoon J. W. Leslie E. J. Kietzman H. W. Ansen-Wilson L. J. (2017). Sonic hedgehog regulation of Foxf2 promotes cranial neural crest mesenchyme proliferation and is disrupted in cleft lip morphogenesis. Development 144, 2082–2091. 10.1242/dev.149930 28506991 PMC5482988

[B33] Ferretti E. Li B. Zewdu R. Wells V. Hebert J. M. Karner C. (2011). A conserved Pbx-Wnt-p63-Irf6 regulatory module controls face morphogenesis by promoting epithelial apoptosis. Dev. Cell 21, 627–641. 10.1016/j.devcel.2011.08.005 21982646 PMC3199312

[B34] Fitzpatrick D. R. Denhez F. Kondaiah P. Akhurst R. J. (1990). Differential expression of TGF beta isoforms in murine palatogenesis. Development 109, 585–595. 10.1242/dev.109.3.585 2401212

[B35] Fontoura C. Silva R. M. Granjeiro J. M. Letra A. (2015). Association of WNT9B gene polymorphisms with nonsyndromic cleft lip with or without cleft palate in Brazilian nuclear families. Cleft Palate Craniofac J. 52, 44–48. 10.1597/13-146 24437584 PMC4102668

[B36] Foppiano S. Hu D. Marcucio R. S. (2007). Signaling by bone morphogenetic proteins directs formation of an ectodermal signaling center that regulates craniofacial development. Dev. Biol. 312, 103–114. 10.1016/j.ydbio.2007.09.016 18028903 PMC2192628

[B37] Francis-West P. Ladher R. Barlow A. Graveson A. (1998). Signalling interactions during facial development. Mech. Dev. 75, 3–28. 10.1016/s0925-4773(98)00082-3 9739099

[B38] Francis-West P. H. Tatla T. Brickell P. M. (1994). Expression patterns of the bone morphogenetic protein genes Bmp-4 and Bmp-2 in the developing chick face suggest a role in outgrowth of the primordia. Dev. Dyn. 201, 168–178. 10.1002/aja.1002010207 7873788

[B39] Fuchs A. Inthal A. Herrmann D. Cheng S. Nakatomi M. Peters H. (2010). Regulation of Tbx22 during facial and palatal development. Dev. Dyn. 239, 2860–2874. 10.1002/dvdy.22421 20845426

[B40] Funato N. Nakamura M. Richardson J. A. Srivastava D. Yanagisawa H. (2012). Tbx1 regulates oral epithelial adhesion and palatal development. Hum. Mol. Genet. 21, 2524–2537. 10.1093/hmg/dds071 22371266 PMC3607464

[B41] Gaare J. D. Langman J. (1977). Fusion of nasal swellings in the mouse embryo: regression of the nasal fin. Am. J. Anat. 150, 477–499. 10.1002/aja.1001500308 930860

[B42] García-Castro M. I. Marcelle C. Bronner-Fraser M. (2002). Ectodermal Wnt function as a neural crest inducer. Science 297, 848–851. 10.1126/science.1070824 12161657

[B43] Gomez G. A. Prasad M. S. Sandhu N. Shelar P. B. Leung A. W. García-Castro M. I. (2019). Human neural crest induction by temporal modulation of WNT activation. Dev. Biol. 449, 99–106. 10.1016/j.ydbio.2019.02.015 30826399 PMC6685424

[B44] Goudy S. Law A. Sanchez G. Baldwin H. S. Brown C. (2010). Tbx1 is necessary for palatal elongation and elevation. Mech. Dev. 127, 292–300. 10.1016/j.mod.2010.03.001 20214979 PMC2871954

[B45] Gritli-Linde A. (2007). Molecular control of secondary palate development. Dev. Biol. 301, 309–326. 10.1016/j.ydbio.2006.07.042 16942766

[B46] Gritli-Linde A. (2008). The etiopathogenesis of cleft lip and cleft palate: usefulness and caveats of mouse models. Curr. Top. Dev. Biol. 84, 37–138. 10.1016/S0070-2153(08)00602-9 19186243

[B47] Gritli-Linde A. (2012). The mouse as a developmental model for cleft lip and palate research. Front. Oral Biol. 16, 32–51. 10.1159/000337523 22759668

[B48] Hammond N. L. Dixon J. Dixon M. J. (2019). Periderm: life-cycle and function during orofacial and epidermal development. Semin. Cell Dev. Biol. 91, 75–83. 10.1016/j.semcdb.2017.08.021 28803895

[B49] Hammond N. L. Dixon M. J. (2022). Revisiting the embryogenesis of lip and palate development. Oral Dis. 28, 1306–1326. 10.1111/odi.14174 35226783 PMC10234451

[B50] Han J. Mayo J. Xu X. Li J. Bringas P. Jr. Maas R. L. (2009). Indirect modulation of Shh signaling by Dlx5 affects the oral-nasal patterning of palate and rescues cleft palate in Msx1-null mice. Development 136, 4225–4233. 10.1242/dev.036723 19934017 PMC2781056

[B51] He F. Xiong W. Wang Y. Matsui M. Yu X. Chai Y. (2010). Modulation of BMP signaling by Noggin is required for the maintenance of palatal epithelial integrity during palatogenesis. Dev. Biol. 347, 109–121. 10.1016/j.ydbio.2010.08.014 20727875 PMC3010875

[B52] He F. Xiong W. Yu X. Espinoza-Lewis R. Liu C. Gu S. (2008). Wnt5a regulates directional cell migration and cell proliferation via Ror2-mediated noncanonical pathway in mammalian palate development. Development 135, 3871–3879. 10.1242/dev.025767 18948417 PMC3010758

[B53] Higashihori N. Buchtová M. Richman J. M. (2010). The function and regulation of TBX22 in avian frontonasal morphogenesis. Dev. Dyn. 239, 458–473. 10.1002/dvdy.22182 20033915

[B54] Hilliard S. A. Yu L. Gu S. Zhang Z. Chen Y. P. (2005). Regional regulation of palatal growth and patterning along the anterior-posterior axis in mice. J. Anat. 207, 655–667. 10.1111/j.1469-7580.2005.00474.x 16313398 PMC1571556

[B55] Hoover F. Glover J. C. (1998). Regional pattern of retinoid X receptor-alpha gene expression in the central nervous system of the chicken embryo and its up-regulation by exposure to 9-cis retinoic acid. J. Comp. Neurol. 398, 575–586. 10.1002/(sici)1096-9861(19980907)398:4<575::aid-cne9>3.0.co;2-# 9717711

[B56] Hu D. Helms J. A. (1999). The role of sonic hedgehog in normal and abnormal craniofacial morphogenesis. Development 126, 4873–4884. 10.1242/dev.126.21.4873 10518503

[B57] Hu D. Marcucio R. S. (2009a). A SHH-responsive signaling center in the forebrain regulates craniofacial morphogenesis via the facial ectoderm. Development 136, 107–116. 10.1242/dev.026583 19036802 PMC2685963

[B58] Hu D. Marcucio R. S. (2009b). Unique organization of the frontonasal ectodermal zone in birds and mammals. Dev. Biol. 325, 200–210. 10.1016/j.ydbio.2008.10.026 19013147 PMC2662765

[B59] Hu D. Marcucio R. S. (2012). Neural crest cells pattern the surface cephalic ectoderm during FEZ formation. Dev. Dyn. 241, 732–740. 10.1002/dvdy.23764 22411554 PMC3422019

[B60] Hu D. Marcucio R. S. Helms J. A. (2003). A zone of frontonasal ectoderm regulates patterning and growth in the face. Development 130, 1749–1758. 10.1242/dev.00397 12642481

[B61] Hu D. Young N. M. Li X. Xu Y. Hallgrímsson B. Marcucio R. S. (2015). A dynamic Shh expression pattern, regulated by SHH and BMP signaling, coordinates fusion of primordia in the amniote face. Development 142, 567–574. 10.1242/dev.114835 25605783 PMC4302993

[B62] Ingham P. W. Mcmahon A. P. (2001). Hedgehog signaling in animal development: paradigms and principles. Genes Dev. 15, 3059–3087. 10.1101/gad.938601 11731473

[B63] Ingraham C. R. Kinoshita A. Kondo S. Yang B. Sajan S. Trout K. J. (2006). Abnormal skin, limb and craniofacial morphogenesis in mice deficient for interferon regulatory factor 6 (Irf6). Nat. Genet. 38, 1335–1340. 10.1038/ng1903 17041601 PMC2082114

[B64] Jeong J. Mao J. Tenzen T. Kottmann A. H. Mcmahon A. P. (2004). Hedgehog signaling in the neural crest cells regulates the patterning and growth of facial primordia. Genes Dev. 18, 937–951. 10.1101/gad.1190304 15107405 PMC395852

[B65] Ji Y. Hao H. Reynolds K. Mcmahon M. Zhou C. J. (2019). Wnt signaling in neural crest ontogenesis and oncogenesis. Cells 8, 1173. 10.3390/cells8101173 31569501 PMC6829301

[B66] Jiang R. Bush J. O. Lidral A. C. (2006). Development of the upper lip: morphogenetic and molecular mechanisms. Dev. Dyn. 235, 1152–1166. 10.1002/dvdy.20646 16292776 PMC2562450

[B67] Jin J. Z. Ding J. (2006). Analysis of cell migration, transdifferentiation and apoptosis during mouse secondary palate fusion. Development 133, 3341–3347. 10.1242/dev.02520 16887819

[B68] Jin Y. R. Han X. H. Taketo M. M. Yoon J. K. (2012). Wnt9b-dependent FGF signaling is crucial for outgrowth of the nasal and maxillary processes during upper jaw and lip development. Development 139, 1821–1830. 10.1242/dev.075796 22461561 PMC3328181

[B69] Jones N. C. Trainor P. A. (2004). The therapeutic potential of stem cells in the treatment of craniofacial abnormalities. Expert Opin. Biol. Ther. 4, 645–657. 10.1517/14712598.4.5.645 15155156

[B70] Kist R. Greally E. Peters H. (2007). Derivation of a mouse model for conditional inactivation of Pax9. Genesis 45, 460–464. 10.1002/dvg.20295 17610273

[B71] Knecht A. K. Bronner-Fraser M. (2002). Induction of the neural crest: a multigene process. Nat. Rev. Genet. 3, 453–461. 10.1038/nrg819 12042772

[B72] Kousa Y. A. Schutte B. C. (2016). Toward an orofacial gene regulatory network. Dev. Dyn. 245, 220–232. 10.1002/dvdy.24341 26332872 PMC4755791

[B73] Kurosaka H. (2015). The roles of hedgehog signaling in upper lip formation. Biomed. Res. Int. 2015, 901041. 10.1155/2015/901041 26425560 PMC4573885

[B74] Kurosaka H. Iulianella A. Williams T. Trainor P. A. (2014). Disrupting hedgehog and WNT signaling interactions promotes cleft lip pathogenesis. J. Clin. Invest 124, 1660–1671. 10.1172/JCI72688 24590292 PMC3973078

[B75] Lan Y. Jiang R. (2009). Sonic hedgehog signaling regulates reciprocal epithelial-mesenchymal interactions controlling palatal outgrowth. Development 136, 1387–1396. 10.1242/dev.028167 19304890 PMC2687468

[B76] Lan Y. Jiang R. (2022). Mouse models in palate development and orofacial cleft research: understanding the crucial role and regulation of epithelial integrity in facial and palate morphogenesis. Curr. Top. Dev. Biol. 148, 13–50. 10.1016/bs.ctdb.2021.12.003 35461563 PMC9060390

[B77] Lan Y. Kingsley P. D. Cho E. S. Jiang R. (2001). Osr2, a new mouse gene related to Drosophila odd-skipped, exhibits dynamic expression patterns during craniofacial, limb, and kidney development. Mech. Dev. 107, 175–179. 10.1016/s0925-4773(01)00457-9 11520675

[B78] Lan Y. Ryan R. C. Zhang Z. Bullard S. A. Bush J. O. Maltby K. M. (2006). Expression of Wnt9b and activation of canonical Wnt signaling during midfacial morphogenesis in mice. Dev. Dyn. 235, 1448–1454. 10.1002/dvdy.20723 16496313 PMC2559872

[B79] Lee J. M. Kim J. Y. Cho K. W. Lee M. J. Cho S. W. Kwak S. (2008). Wnt11/Fgfr1b cross-talk modulates the fate of cells in palate development. Dev. Biol. 314, 341–350. 10.1016/j.ydbio.2007.11.033 18191119

[B80] Li C. Lan Y. Jiang R. (2017). Molecular and cellular mechanisms of palate development. J. Dent. Res. 96, 1184–1191. 10.1177/0022034517703580 28745929 PMC5613875

[B81] Li L. Shi J. Y. Zhu G. Q. Shi B. (2012). MiR-17-92 cluster regulates cell proliferation and collagen synthesis by targeting TGFB pathway in mouse palatal mesenchymal cells. J. Cell Biochem. 113, 1235–1244. 10.1002/jcb.23457 22095742

[B82] Liem K. F. Jr. Tremml G. Roelink H. Jessell T. M. (1995). Dorsal differentiation of neural plate cells induced by BMP-mediated signals from epidermal ectoderm. Cell 82, 969–979. 10.1016/0092-8674(95)90276-7 7553857

[B83] Lin C. Fisher A. V. Yin Y. Maruyama T. Veith G. M. Dhandha M. (2011). The inductive role of Wnt-β-Catenin signaling in the formation of oral apparatus. Dev. Biol. 356, 40–50. 10.1016/j.ydbio.2011.05.002 21600200 PMC3130801

[B84] Liu P. Wakamiya M. Shea M. J. Albrecht U. Behringer R. R. Bradley A. (1999). Requirement for Wnt3 in vertebrate axis formation. Nat. Genet. 22, 361–365. 10.1038/11932 10431240

[B85] Liu W. Sun X. Braut A. Mishina Y. Behringer R. R. Mina M. (2005). Distinct functions for Bmp signaling in lip and palate fusion in mice. Development 132, 1453–1461. 10.1242/dev.01676 15716346

[B86] Liu X. Zhang H. Gao L. Yin Y. Pan X. Li Z. (2014). Negative interplay of retinoic acid and TGF-β signaling mediated by TG-interacting factor to modulate mouse embryonic palate mesenchymal-cell proliferation. Birth Defects Res. B Dev. Reprod. Toxicol. 101, 403–409. 10.1002/bdrb.21130 25477235

[B87] Liu Y. Wang M. Zhao W. Yuan X. Yang X. Li Y. (2015). Gpr177-mediated Wnt signaling is required for secondary palate development. J. Dent. Res. 94, 961–967. 10.1177/0022034515583532 25922332

[B88] Lu Y. P. Han W. T. Liu Q. Li J. X. Li Z. J. Jiang M. (2015). Variations in WNT3 gene are associated with incidence of non-syndromic cleft lip with or without cleft palate in a northeast Chinese population. Genet. Mol. Res. 14, 12646–12653. 10.4238/2015.October.19.8 26505415

[B89] Marchant L. Linker C. Ruiz P. Guerrero N. Mayor R. (1998). The inductive properties of mesoderm suggest that the neural crest cells are specified by a BMP gradient. Dev. Biol. 198, 319–329. 10.1016/s0012-1606(98)80008-0 9659936

[B90] Marchini M. Hu D. Lo Vercio L. Young N. M. Forkert N. D. Hallgrímsson B. (2021). Wnt signaling drives correlated changes in facial morphology and brain shape. Front. Cell Dev. Biol. 9, 644099. 10.3389/fcell.2021.644099 33855022 PMC8039397

[B91] Martínez-Alvarez C. Bonelli R. Tudela C. Gato A. Mena J. O'kane S. (2000). Bulging medial edge epithelial cells and palatal fusion. Int. J. Dev. Biol. 44, 331–335. 10853831

[B92] Martínez-Morales P. L. Diez Del Corral R. Olivera-Martínez I. Quiroga A. C. Das R. M. Barbas J. A. (2011). FGF and retinoic acid activity gradients control the timing of neural crest cell emigration in the trunk. J. Cell Biol. 194, 489–503. 10.1083/jcb.201011077 21807879 PMC3153641

[B93] Massari M. E. Murre C. (2000). Helix-loop-helix proteins: regulators of transcription in eucaryotic organisms. Mol. Cell Biol. 20, 429–440. 10.1128/mcb.20.2.429-440.2000 10611221 PMC85097

[B94] Mcgonnell I. M. Clarke J. D. Tickle C. (1998). Fate map of the developing chick face: analysis of expansion of facial primordia and establishment of the primary palate. Dev. Dyn. official Publ. Am. Assoc. Anatomists 212, 102–118. 10.1002/(SICI)1097-0177(199805)212:1<102::AID-AJA10>3.0.CO;2-9 9603428

[B95] Metzis V. Courtney A. D. Kerr M. C. Ferguson C. Rondón Galeano M. C. Parton R. G. (2013). Patched1 is required in neural crest cells for the prevention of orofacial clefts. Hum. Mol. Genet. 22, 5026–5035. 10.1093/hmg/ddt353 23900075

[B96] Minoux M. Rijli F. M. (2010). Molecular mechanisms of cranial neural crest cell migration and patterning in craniofacial development. Development 137, 2605–2621. 10.1242/dev.040048 20663816

[B97] Monsoro-Burq A. H. Fletcher R. B. Harland R. M. (2003). Neural crest induction by paraxial mesoderm in Xenopus embryos requires FGF signals. Development 130, 3111–3124. 10.1242/dev.00531 12783784

[B98] Moretti F. Marinari B. Lo Iacono N. Botti E. Giunta A. Spallone G. (2010). A regulatory feedback loop involving p63 and IRF6 links the pathogenesis of 2 genetically different human ectodermal dysplasias. J. Clin. Invest 120, 1570–1577. 10.1172/JCI40267 20424325 PMC2860936

[B99] Morikawa Y. D'autréaux F. Gershon M. D. Cserjesi P. (2007). Hand2 determines the noradrenergic phenotype in the mouse sympathetic nervous system. Dev. Biol. 307, 114–126. 10.1016/j.ydbio.2007.04.027 17531968 PMC1952239

[B100] Mossey P. Modell B. (2012). Epidemiology of oral clefts 2012: an international perspective. Cleft lip palate 16, 1–18. 10.1159/000337464 22759666

[B101] Mossey P. A. Little J. Munger R. G. Dixon M. J. Shaw W. C. (2009). Cleft lip and palate. Lancet 374, 1773–1785. 10.1016/S0140-6736(09)60695-4 19747722

[B102] Mostowska A. Hozyasz K. K. Biedziak B. Wojcicki P. Lianeri M. Jagodzinski P. P. (2012). Genotype and haplotype analysis of WNT genes in non-syndromic cleft lip with or without cleft palate. Eur. J. Oral Sci. 120, 1–8. 10.1111/j.1600-0722.2011.00938.x 22288914

[B103] Nakajima A. C F. S. Gulka A. O. D. Hanai J. I. (2018). TGF-Β signaling and the epithelial-mesenchymal transition during palatal fusion. Int. J. Mol. Sci. 19, 3638. 10.3390/ijms19113638 30463190 PMC6274911

[B104] Nik A. M. Johansson J. A. Ghiami M. Reyahi A. Carlsson P. (2016). Foxf2 is required for secondary palate development and Tgfβ signaling in palatal shelf mesenchyme. Dev. Biol. 415, 14–23. 10.1016/j.ydbio.2016.05.013 27180663

[B105] Odent S. Atti-Bitach T. Blayau M. Mathieu M. Aug J. Delezo De A. L. (1999). Expression of the Sonic hedgehog (SHH) gene during early human development and phenotypic expression of new mutations causing holoprosencephaly. Hum. Mol. Genet. 8, 1683–1689. 10.1093/hmg/8.9.1683 10441331

[B106] Okano J. Kimura W. Papaionnou V. E. Miura N. Yamada G. Shiota K. (2012). The regulation of endogenous retinoic acid level through CYP26B1 is required for elevation of palatal shelves. Dev. Dyn. 241, 1744–1756. 10.1002/dvdy.23862 22972661

[B107] O'rahilly R. Müller F. (2007). The development of the neural crest in the human. J. Anat. 211, 335–351. 10.1111/j.1469-7580.2007.00773.x 17848161 PMC2375817

[B108] Parada C. Chai Y. (2012). Roles of BMP signaling pathway in lip and palate development. Front. Oral Biol. 16, 60–70. 10.1159/000337617 22759670 PMC3661199

[B109] Piacentino M. L. Hutchins E. J. Bronner M. E. (2021). Essential function and targets of BMP signaling during midbrain neural crest delamination. Dev. Biol. 477, 251–261. 10.1016/j.ydbio.2021.06.003 34102166 PMC8277753

[B110] Proetzel G. Pawlowski S. A. Wiles M. V. Yin M. Boivin G. P. Howles P. N. (1995). Transforming growth factor-beta 3 is required for secondary palate fusion. Nat. Genet. 11, 409–414. 10.1038/ng1295-409 7493021 PMC3855390

[B111] Reid B. S. Yang H. Melvin V. S. Taketo M. M. Williams T. (2011). Ectodermal Wnt/β-catenin signaling shapes the mouse face. Dev. Biol. 349, 261–269. 10.1016/j.ydbio.2010.11.012 21087601 PMC3057077

[B112] Rekler D. Kalcheim C. (2022). Completion of neural crest cell production and emigration is regulated by retinoic-acid-dependent inhibition of BMP signaling. Elife 11, e72723. 10.7554/eLife.72723 35394423 PMC8993216

[B113] Reynolds K. Kumari P. Sepulveda Rincon L. Gu R. Ji Y. Kumar S. (2019). Wnt signaling in orofacial clefts: crosstalk, pathogenesis and models. Dis. Model Mech. 12, dmm037051. 10.1242/dmm.037051 30760477 PMC6398499

[B114] Reynolds K. Zhang S. Sun B. Garland M. A. Ji Y. Zhou C. J. (2020). Genetics and signaling mechanisms of orofacial clefts. Birth defects Res. 112, 1588–1634. 10.1002/bdr2.1754 32666711 PMC7883771

[B115] Ribatti D. Santoiemma M. (2014). Epithelial-mesenchymal interactions: a fundamental Developmental Biology mechanism. Int. J. Dev. Biol. 58, 303–306. 10.1387/ijdb.140143dr 25354449

[B116] Rice R. Spencer-Dene B. Connor E. C. Gritli-Linde A. Mcmahon A. P. Dickson C. (2004). Disruption of Fgf10/Fgfr2b-coordinated epithelial-mesenchymal interactions causes cleft palate. J. Clin. Invest 113, 1692–1700. 10.1172/JCI20384 15199404 PMC420504

[B117] Richardson R. Mitchell K. Hammond N. L. Mollo M. R. Kouwenhoven E. N. Wyatt N. D. (2017). p63 exerts spatio-temporal control of palatal epithelial cell fate to prevent cleft palate. PLoS Genet. 13, e1006828. 10.1371/journal.pgen.1006828 28604778 PMC5484519

[B118] Richman J. M. Tickle C. (1989). Epithelia are interchangeable between facial primordia of chick embryos and morphogenesis is controlled by the mesenchyme. Dev. Biol. 136, 201–210. 10.1016/0012-1606(89)90142-5 2806720

[B119] Roth D. M. Bayona F. Baddam P. Graf D. (2021). Craniofacial development: neural crest in molecular embryology. Head Neck Pathology 15, 1–15. 10.1007/s12105-021-01301-z 33723764 PMC8010074

[B120] Rowe A. Brickell P. M. (1995). Expression of the chicken retinoid X receptor-gamma gene in migrating cranial neural crest cells. Anat. Embryol. Berl. 192, 1–8. 10.1007/BF00186986 7485997

[B121] Rowe A. Richman J. M. Brickell P. M. (1992). Development of the spatial pattern of retinoic acid receptor-beta transcripts in embryonic chick facial primordia. Development 114, 805–813. 10.1242/dev.114.3.805 1319895

[B122] Ruest L. B. Dager M. Yanagisawa H. Charité J. Hammer R. E. Olson E. N. (2003). dHAND-Cre transgenic mice reveal specific potential functions of dHAND during craniofacial development. Dev. Biol. 257, 263–277. 10.1016/s0012-1606(03)00068-x 12729557 PMC2830752

[B123] Saleem K. Zaib T. Sun W. Fu S. (2019). Assessment of candidate genes and genetic heterogeneity in human non syndromic orofacial clefts specifically non syndromic cleft lip with or without palate. Heliyon 5, e03019. 10.1016/j.heliyon.2019.e03019 31886431 PMC6921104

[B124] Sasaki Y. O'kane S. Dixon J. Dixon M. J. Ferguson M. W. (2007). Temporal and spatial expression of Pax9 and Sonic hedgehog during development of normal mouse palates and cleft palates in TGF-beta3 null embryos. Arch. Oral Biol. 52, 260–267. 10.1016/j.archoralbio.2006.09.012 17097601

[B125] Sauka-Spengler T. Bronner-Fraser M. (2008). A gene regulatory network orchestrates neural crest formation. Nat. Rev. Mol. Cell Biol. 9, 557–568. 10.1038/nrm2428 18523435

[B126] Schneider R. A. Hu D. Rubenstein J. L. Maden M. Helms J. A. (2001). Local retinoid signaling coordinates forebrain and facial morphogenesis by maintaining FGF8 and SHH. Development 128, 2755–2767. 10.1242/dev.128.14.2755 11526081

[B127] Shawber C. Boulter J. Lindsell C. E. Weinmaster G. (1996). Jagged2: a serrate-like gene expressed during rat embryogenesis. Dev. Biol. 180, 370–376. 10.1006/dbio.1996.0310 8948600

[B128] Shigetani Y. Nobusada Y. Kuratani S. (2000). Ectodermally derived FGF8 defines the maxillomandibular region in the early chick embryo: epithelial-mesenchymal interactions in the specification of the craniofacial ectomesenchyme. Dev. Biol. 228, 73–85. 10.1006/dbio.2000.9932 11087627

[B129] Silver M. H. Foidart J. M. Pratt R. M. (1981). Distribution of fibronectin and collagen during mouse limb and palate development. Differentiation 18, 141–149. 10.1111/j.1432-0436.1981.tb01115.x 7035260

[B130] Simões-Costa M. Bronner M. E. (2015). Establishing neural crest identity: a gene regulatory recipe. Development 142, 242–257. 10.1242/dev.105445 25564621 PMC4302844

[B131] Soldatov R. Kaucka M. Kastriti M. E. Petersen J. Chontorotzea T. Englmaier L. (2019). Spatiotemporal structure of cell fate decisions in murine neural crest. Science 364, eaas9536. 10.1126/science.aas9536 31171666

[B132] Song L. Li Y. Wang K. Wang Y. Z. Molotkov A. Gao L. (2009). Lrp6-mediated canonical Wnt signaling is required for lip formation and fusion. Development 136, 3161–3171. 10.1242/dev.037440 19700620

[B133] Sull J. W. Liang K. Y. Hetmanski J. B. Wu T. Fallin M. D. Ingersoll R. G. (2009). Evidence that TGFA influences risk to cleft lip with/without cleft palate through unconventional genetic mechanisms. Hum. Genet. 126, 385–394. 10.1007/s00439-009-0680-3 19444471 PMC2901599

[B134] Sun D. Baur S. Hay E. D. (2000). Epithelial-mesenchymal transformation is the mechanism for fusion of the craniofacial primordia involved in morphogenesis of the chicken lip. Dev. Biol. 228, 337–349. 10.1006/dbio.2000.9946 11112334

[B135] Tamarin A. Boyde A. (1977). Facial and visceral arch development in the mouse embryo: a study by scanning electron microscopy. J. Anat. 124, 563–580. 604328 PMC1234654

[B136] Taya Y. O'kane S. Ferguson M. W. (1999). Pathogenesis of cleft palate in TGF-beta3 knockout mice. Development 126, 3869–3879. 10.1242/dev.126.17.3869 10433915

[B137] Teshima T. H. Lourenco S. V. Tucker A. S. (2016). Multiple cranial organ defects after conditionally knocking out Fgf10 in the neural crest. Front. Physiol. 7, 488. 10.3389/fphys.2016.00488 27826253 PMC5078472

[B138] Thomason H. A. Dixon M. J. Dixon J. (2008). Facial clefting in Tp63 deficient mice results from altered Bmp4, Fgf8 and Shh signaling. Dev. Biol. 321, 273–282. 10.1016/j.ydbio.2008.06.030 18634775

[B139] Thomason H. A. Zhou H. Kouwenhoven E. N. Dotto G. P. Restivo G. Nguyen B. C. (2010). Cooperation between the transcription factors p63 and IRF6 is essential to prevent cleft palate in mice. J. Clin. Invest 120, 1561–1569. 10.1172/JCI40266 20424327 PMC2860913

[B140] Tribulo C. Aybar M. J. Nguyen V. H. Mullins M. C. Mayor R. (2003). Regulation of Msx genes by a Bmp gradient is essential for neural crest specification. Development 130, 6441–6452. 10.1242/dev.00878 14627721

[B141] Trousse F. Esteve P. Bovolenta P. (2001). Bmp4 mediates apoptotic cell death in the developing chick eye. J. Neurosci. 21, 1292–1301. 10.1523/JNEUROSCI.21-04-01292.2001 11160400 PMC6762245

[B142] Vaziri Sani F. Kaartinen V. El Shahawy M. Linde A. Gritli-Linde A. (2010). Developmental changes in cellular and extracellular structural macromolecules in the secondary palate and in the nasal cavity of the mouse. Eur. J. Oral Sci. 118, 221–236. 10.1111/j.1600-0722.2010.00732.x 20572855

[B143] Wan M. Cao X. (2005). BMP signaling in skeletal development. Biochem. Biophys. Res. Commun. 328, 651–657. 10.1016/j.bbrc.2004.11.067 15694398

[B144] Wang R. N. Green J. Wang Z. Deng Y. Qiao M. Peabody M. (2014). Bone Morphogenetic Protein (BMP) signaling in development and human diseases. Genes Dis. 1, 87–105. 10.1016/j.gendis.2014.07.005 25401122 PMC4232216

[B145] Wang X. Li C. Zhu Z. Yuan L. Chan W. Y. Sha O. (2020). Extracellular matrix remodeling during palate development. Organogenesis 16, 43–60. 10.1080/15476278.2020.1735239 32233728 PMC7531623

[B146] Wang Y. Dai Y. Li X. Chen C. Y. Li W. Yu Z. (2011a). Inhibition of Smad signaling is implicated in cleft palate induced by all-trans retinoic acid. Acta Biol. Hung 62, 142–150. 10.1556/ABiol.62.2011.2.4 21555266

[B147] Wang Y. Song L. Zhou C. J. (2011b). The canonical Wnt/β-catenin signaling pathway regulates Fgf signaling for early facial development. Dev. Biol. 349, 250–260. 10.1016/j.ydbio.2010.11.004 21070765

[B148] White J. A. Beckett-Jones B. Guo Y. D. Dilworth F. J. Bonasoro J. Jones G. (1997). cDNA cloning of human retinoic acid-metabolizing enzyme (hP450RAI) identifies a novel family of cytochromes P450. J. Biol. Chem. 272, 18538–18541. 10.1074/jbc.272.30.18538 9228017

[B149] Wilson P. A. Lagna G. Suzuki A. Hemmati-Brivanlou A. (1997). Concentration-dependent patterning of the Xenopus ectoderm by BMP4 and its signal transducer Smad1. Development 124, 3177–3184. 10.1242/dev.124.16.3177 9272958

[B150] Won H.-J. Kim J.-W. Won H.-S. Shin J.-O. (2023). Gene regulatory networks and signaling pathways in palatogenesis and cleft palate: a comprehensive review. Cells 12, 1954. 10.3390/cells12151954 37566033 PMC10416829

[B151] Wu W. Gu S. Sun C. He W. Xie X. Li X. (2015). Altered FGF signaling pathways impair cell proliferation and elevation of palate shelves. PLoS One 10, e0136951. 10.1371/journal.pone.0136951 26332583 PMC4558018

[B152] Xiong W. He F. Morikawa Y. Yu X. Zhang Z. Lan Y. (2009). Hand2 is required in the epithelium for palatogenesis in mice. Dev. Biol. 330, 131–141. 10.1016/j.ydbio.2009.03.021 19341725 PMC2745957

[B153] Xu J. Liu H. Lan Y. Aronow B. J. Kalinichenko V. V. Jiang R. (2016). A shh-foxf-fgf18-shh molecular circuit regulating palate development. PLoS Genet. 12, e1005769. 10.1371/journal.pgen.1005769 26745863 PMC4712829

[B154] Xu Q. Jamniczky H. Hu D. Green R. M. Marcucio R. S. Hallgrimsson B. (2015). Correlations between the morphology of sonic hedgehog expression domains and embryonic craniofacial shape. Evol. Biol. 42, 379–386. 10.1007/s11692-015-9321-z 26321772 PMC4548935

[B155] Xu X. Han J. Ito Y. Bringas P. Jr. Urata M. M. Chai Y. (2006). Cell autonomous requirement for Tgfbr2 in the disappearance of medial edge epithelium during palatal fusion. Dev. Biol. 297, 238–248. 10.1016/j.ydbio.2006.05.014 16780827

[B156] Yan F. Dai Y. Iwata J. Zhao Z. Jia P. (2020). An integrative, genomic, transcriptomic and network-assisted study to identify genes associated with human cleft lip with or without cleft palate. BMC Med. Genomics 13, 39. 10.1186/s12920-020-0675-4 32241273 PMC7118807

[B157] Yang A. Zhu Z. Kapranov P. Mckeon F. Church G. M. Gingeras T. R. (2006). Relationships between p63 binding, DNA sequence, transcription activity, and biological function in human cells. Mol. Cell 24, 593–602. 10.1016/j.molcel.2006.10.018 17188034

[B158] Yoshida T. Vivatbutsiri P. Morriss-Kay G. Saga Y. Iseki S. (2008). Cell lineage in mammalian craniofacial mesenchyme. Mech. Dev. 125, 797–808. 10.1016/j.mod.2008.06.007 18617001

[B159] Yu K. Deng M. Naluai-Cecchini T. Glass I. A. Cox T. C. (2017). Differences in oral structure and tissue interactions during mouse vs. Human palatogenesis: implications for the translation of findings from mice. Front. Physiol. 8, 154. 10.3389/fphys.2017.00154 28360863 PMC5350148

[B160] Yu W. Ruest L. B. Svoboda K. K. (2009). Regulation of epithelial-mesenchymal transition in palatal fusion. Exp. Biol. Med. (Maywood) 234, 483–491. 10.3181/0812-MR-365 19234053

[B161] Yu Z. Lin J. Xiao Y. Han J. Zhang X. Jia H. (2005). Induction of cell-cycle arrest by all-trans retinoic acid in mouse embryonic palatal mesenchymal (MEPM) cells. Toxicol. Sci. 83, 349–354. 10.1093/toxsci/kfi030 15537748

[B162] Yu Z. Xing Y. (2006). atRA-induced apoptosis of mouse embryonic palate mesenchymal cells involves activation of MAPK pathway. Toxicol. Appl. Pharmacol. 215, 57–63. 10.1016/j.taap.2006.04.001 16712891

[B163] Zhang Z. Song Y. Zhao X. Zhang X. Fermin C. Chen Y. (2002). Rescue of cleft palate in Msx1-deficient mice by transgenic Bmp4 reveals a network of BMP and Shh signaling in the regulation of mammalian palatogenesis. Development 129, 4135–4146. 10.1242/dev.129.17.4135 12163415

[B164] Zhou J. Gao Y. Lan Y. Jia S. Jiang R. (2013). Pax9 regulates a molecular network involving Bmp4, Fgf10, Shh signaling and the Osr2 transcription factor to control palate morphogenesis. Development 140, 4709–4718. 10.1242/dev.099028 24173808 PMC3833429

[B165] Zhu X. J. Liu Y. Yuan X. Wang M. Zhao W. Yang X. (2016). Ectodermal Wnt controls nasal pit morphogenesis through modulation of the BMP/FGF/JNK signaling axis. Dev. Dyn. 245, 414–426. 10.1002/dvdy.24376 26661618 PMC4774528

[B166] Zoupa M. Xavier G. M. Bryan S. Theologidis I. Arno M. Cobourne M. T. (2018). Gene expression profiling in the developing secondary palate in the absence of Tbx1 function. BMC Genomics 19, 429. 10.1186/s12864-018-4782-y 29866044 PMC5987606

